# Multidrug-Resistant *Escherichia coli* Strains to Last Resort Human Antibiotics Isolated from Healthy Companion Animals in Valencia Region

**DOI:** 10.3390/antibiotics12111638

**Published:** 2023-11-19

**Authors:** Ana Marco-Fuertes, Jaume Jordá, Clara Marin, Laura Lorenzo-Rebenaque, Laura Montoro-Dasi, Santiago Vega

**Affiliations:** 1 Departamento de Producción y Sanidad Animal, Salud Pública Veterinaria y Ciencia y Tecnología de los Alimentos, Facultad de Veterinaria, Instituto de Ciencias Biomédicas, Universidad Cardenal Herrera-CEU, CEU Universities, Calle Santiago Ramón y Cajal 20, Alfara del Patriarca, 45115 Valencia, Spain; ana.marcofuertes@uchceu.es (A.M.-F.); jaume.jorda@uchceu.es (J.J.); clara.marin@uchceu.es (C.M.); svega@uchceu.es (S.V.); 2 Institute of Animal Science and Technology, Universitat Politècnica de València, 46022 Valencia, Spain; laulore@upv.es

**Keywords:** antimicrobial resistance, cats, commensal bacteria, dogs, one health

## Abstract

Failure in antibiotic therapies due to the increase in antimicrobial-resistant (AMR) bacteria is one of the main threats to public and animal health. In recent decades, the perception of companion animals has changed, from being considered as a work tool to a household member, creating a family bond and sharing spaces in their daily routine. Hence, the aim of this study is to assess the current epidemiological situation regarding the presence of AMR and multidrug resistance (MDR) in companion animals in the Valencia Region, using the indicator bacteria *Escherichia coli* as a sentinel. For this purpose, 244 samples of dogs and cats were collected from veterinary centres to assess antimicrobial susceptibility against a panel of 22 antibiotics with public health relevance. A total of 197 *E. coli* strains were isolated from asymptomatic dogs and cats. The results showed AMR against all the 22 antibiotics studied, including those critically important to human medicine. Moreover, almost 50% of the strains presented MDR. The present study revealed the importance of monitoring AMR and MDR trends in companion animals, as they could pose a risk due to the spread of AMR and its resistance genes to humans, other animals and the environment they cohabit.

## 1. Introduction

The rise of AMR bacteria in both humans and animals leads to the failure of antibiotic therapies, posing a major threat to public and animal health. Thus, the World Health Organisation (WHO) includes antimicrobial resistance (AMR) among the top 10 threats to global public health [1]. AMR arises when microorganisms, such as bacteria, viruses or parasites, change over time and become unresponsive to the drugs used to fight the infections they cause, making them difficult to treat and increasing the risk of spreading disease, developing severe forms of illness and death [1].

It is currently estimated that there are more than 340 million companion animals in European households, mainly 127 million cats and 104 million dogs, representing 70% of all companion animals in Europe [2]. In recent decades, there has been a paradigm shift, as the general perception of companion animals has changed from being seen as a work tool to a family member, creating a bond with their owners and providing companionship, and sharing spaces and their daily routine [3,4]. However, these animals not only live with their owners in their homes but also interact with other animals, domestic or wild [5], and people, such as children or the elderly, in public places such as parks, streets or beaches [6]. Moreover, these interaction spaces are increased by allowing their presence in planes, trains and restaurants, etc. This is of vital importance, as humans nowadays maintain intimate contact with their companion animals, increasing the possibility of sharing the microbiota, and its AMR genes [7], which could be shared between humans and animals through the environment they cohabit [3,8,9]. Therefore, it is necessary to encompass this issue under the One Health strategy, which seeks collaboration between healthcare professionals in all aspects of health including humans, animals and the environment [10]. 

Historically, the acquisition of multidrug resistances (MDR), defined as being caused by microorganisms that are resistant to three or more antimicrobials commonly used in the treatment of infections caused by that microorganism, and that this resistance has clinical and epidemiological relevance [11], has been linked to the misuse of antibiotics, particularly in food-producing animals, where they had been widely used as growth promoters [12], and in human health, where antibiotics have been overused [13]. In this context, different surveillance and monitoring programmes have been established in the European Union (EU), both for humans and animals. In human medicine, a European Antimicrobial Resistance Surveillance Network (EARS-Net) has already been established [14,15] to raise awareness of the real problem of AMR in the political, scientific and societal spheres, as well as to promote the implementation, maintenance and improvement in national AMR surveillance programmes to encourage rational use of antibiotics [16]. In addition, also in veterinary medicine, a mandatory monitoring programme for AMR in zoonotic and commensal bacteria was rolled out throughout the EU, coordinated by the European Food Safety Authority (EFSA), focusing particularly on healthy high-production animals such as pigs, chickens and cattle at slaughterhouse level [17].

Moreover, in human medicine, the WHO categorises antimicrobials as ‘critically important’, ‘highly important’ and ‘important’ to human health [18]. In the latest report published in 2023, some antibiotics have changed category, and the main antibiotics authorised for humans and animals are categorised as Critically Important Antimicrobials (CIA) (3rd and 4th gen. cephalosporins, quinolones, polymyxins and phosphonic acid derivatives), whereas antibiotics authorised only for humans were classified as Highest Priority Critical Important Antimicrobial (HPCIA) (including new antibiotic molecules, such as 5th gen. cephalosporins, carbapenems, glycylcyclines or lipopeptides, among others) [18]. Moreover, this current WHO classification matches the one established by the EMA in 2019, used in animals to promote their responsible use in order to protect animal and public health: Category D (“Prudence”), first line of defence; Category C (“Caution”), when Category D antibiotics fail; Category B (“Restrict”), which matches the CIAs, used when all therapeutic alternatives (D and C) have been exhausted; and finally, Category A (“Avoid”), matching the HPCIAs and commonly known as last resort antibiotics, limited to human medicine and not authorised in the EU to treat food-producing animals. However, they can be dispensed in exceptional situations in companion animal clinics, following the prescription order [19]. 

Currently, to complement the present EARS-Net, the EU intends to launch a European Antimicrobial Resistance Surveillance Network in Veterinary Medicine (EARS-Vet), which aims to harmonise existing programmes and AMR surveillance trends in bacteria isolated from diseased animals and include animal species that have not been considered thus far, such as companion animals [20,21]. In Spain, the National Plan against Antibiotic Resistance (PRAN) was adopted in 2014 in response to the European Commission’s Communication of 17 November 2011, setting out an Action Plan on Antimicrobial Resistance, and the Conclusions of the Council of the EU of 29 May 2012 on the impact of antimicrobial resistance and how it should be addressed jointly in human and veterinary health.

The latest animals that are being included in AMR surveillance programmes are companion animals (dogs and cats) [22]. The same applies to surveillance programmes on antimicrobial use (AMU) in companion animals; although some European countries do include these data in their national surveillance programmes [10], there are no total AMU data in the European Medicines Agency (EMA) and World Organisation for Animal Health (OIE) annual reports. This is mainly due to the fact that data on the animal populations of dogs and cats are not available for all participating countries. Nevertheless, from the EMA [23] and OIE [24] available data, penicillins and 1st and 2nd generation cephalosporins, along with tetracyclines, were the most sold and reported classes of antibiotics for companion animals. These results were in line with the AMU reported in some surveillance programmes that included these data, such as the Belgian [25], Danish [26], Norwegian [27], Swedish [28], or United Kingdom [29] programmes, and with the results observed in most studies [30,31,32].

In this context, the importance of monitoring AMR in these animals lies not only in the amount of antibiotics they consume, but in the use of certain antibiotics considered critical for human health reserved to treat MDR hospital-acquired human infections, with the threat this entails to public health. Hence, the aim of this study was to assess the current epidemiological situation regarding the presence of AMR and MDR in companion animals (cats and dogs) in the Valencia Region, using the commensal sentinel bacteria *Escherichia coli* as a model, before setting the objectives in the EU scope.

## 2. Results

In this study, a total of 244 animals were sampled (131 dogs and 113 cats, respectively). Regarding the distribution, 48.4% (70/131 and 48/113, dogs and cats, respectively) of the samples were taken in Veterinary Hospitals, and 51.6% (61/131 and 65/113, dogs and cats, respectively) of the samples were taken in Veterinary Clinics. 

From the collected canine samples, 77.9% (102/131) *E. coli* were isolated. From the samples collected from cats, 84.1% (95/113) *E. coli* were isolated.

### 2.1. Epidemiological Results

From all the animals sampled, a questionnaire with general and clinical information was drawn up in order to study the differences in the appearance of AMR depending on the epidemiological information. 

#### 2.1.1. Dogs

The canine population was distributed by sex, where 53.4% (70/131) were females and 46.6% (61/131) were males. The age of these animals ranged from 1.5 months to 16 years, where 24% (32/131) were puppies, 27% (35/131) were mature dogs, and 49% (64/131) were geriatric dogs. In addition, 45% (59/131) dogs cohabited in their households with other animals. However, all of them (131) went out daily, so they are in close contact with other animals outside the household.

**Figure 1:**
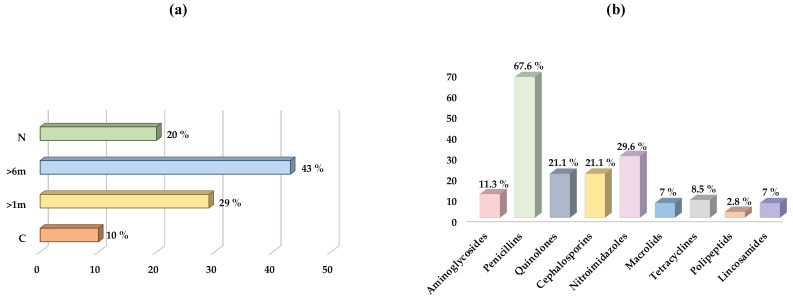
Distribution of the dog population studied, depending on when were they last treated and which antibiotics were the most common. (**a**) Moment of the last antibiotic administration; (**b**) antibiotic groups used in the treatment of infections in the study population at some point of their lives. C: currently. >1m: in the last month. >6m: in the last six months. N: never.

Regarding the clinical data collected from all the dogs sampled, 22.9% (30/131) of the animals presented a chronic disease and 77.1% (101/131) did not present any. The chronic diseases were classified depending on whether they were systemic (76.7%, 23/30) or musculoskeletal (23.3%, 7/30). In addition, regarding the daily medication taken by the animals, 22.1% (29/131) were on some kind of medication. Finally, of all dogs sampled, 83.2% (109/131) had received previous antibiotic therapy at some point in their lives, compared to 16.8% (22/131) that had never been treated with antibiotics. All data on when each animal was last treated with antibiotics and with which group of antibiotics are represented in Figure 1.

#### 2.1.2. Cats

The feline population was distributed by sex, where 55.8% (63/113) were females and 44.2% (50/113) were males. These animals ranged in age from 5 months to 17 years, where 42% (47,113) were kittens, 31% (35/113) were mature cats and 27% (31/113) were geriatric cats. In addition, 69.9% (79/113) of sampled cats cohabited in their households with other animals and only 12.4% (14/113) had contact with other animals outside the home.

**Figure 2:**
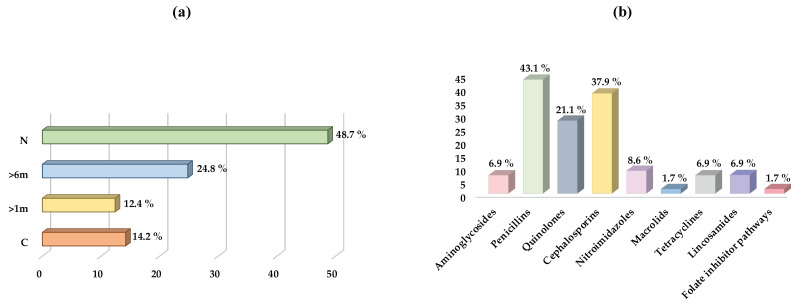
Distribution of the cat population studied, depending on when were they last treated and which antibiotics were the most common. (**a**) Moment of the last antibiotic administration; (**b**) antibiotic groups used in the treatment of infections in the study population at some point of their lives. C: currently. >1m: in the last month. >6m: in the last six months. N: never.

According to the clinical data collected from all the cats sampled, 26.5% (30/113) of the animals presented a chronic disease, all of them classified as systemic diseases, while 73.5% (83/113) did not present any disease. In addition, regarding the daily medication taken by the animals, 12.4% (14/113) were on some kind of medication. Finally, of all cats sampled, 51.3% (58/113) had received previous antibiotic therapy at some point in their lives, compared to 48.7% (55/113) that had never been treated with antibiotics. All data on when each animal was last treated with antibiotics and with which group of antibiotics are represented in Figure 2.

### 2.2. Antimicrobial Susceptibility from E. coli Strains

From all the strains isolated from dogs, 88.2% (90/102) were resistant to at least one of the 22 antimicrobials tested, and 47.1% (48/102) were considered MDR. However, no statistically significant differences were found between the different epidemiological groups described above (*p*-value > 0.05). In contrast, in relation to the strains isolated from cats, lower AMR rates were found than in dogs, where 72.6% (69/95) were resistant to at least one of the antimicrobials tested and 34.7% (33/95) were MDR. In the feline population, no statistically significant differences were found between the different epidemiological groups studied and the occurrence of AMR and MDR (*p*-value > 0.05).

The isolated strains were resistant to at least one of the 22 antimicrobials tested in this study (Table 1). However, of all the antimicrobial groups studied, the penicillins group is the one with the highest AMR, in both dogs and cats (45.8% and 25.5%, respectively) (Table 1). However, when individual AMRs were compared by animal species, the percentages vary (*p*-value > 0.05). For example, the highest frequency of AMR in dogs was found to ampicillin (AMP; 62.7%, 64/102) followed by ticarcillin (TIC; 60.8%, 62/102), whereas in cats, the highest frequency was found against TIC (41.8%, 38/95) followed by AMP (40.7%, 37/91). As for the rest of the antibiotics studied, the differences were more striking, as in the case of amoxicillin/clavulanic acid, (AMC) where there was a huge difference between dogs (43.1%, 44/102) and cats (19%, 17/95), or in the case of gentamicin (GEN), where cat strains (22.6%, 20/95) showed a higher percentage of AMR than dog strains (8.8%, 9/102) (Table 1) (*p*-value > 0.05). Regarding the CIAs (cephalosporins and quinolones) studied, high AMR levels were observed for this antibiotics group (*p*-value < 0.05). Moreover, of all the antibiotics studied, following the WHO categorisation for important antibiotics, four of them belonged to category HPCIAs, as well as to category A according to the EMA categorisation (antibiotics reserved for human treatment only), with a high percentage of AMR being found for this type of antibiotics (Table 1).

Overall, 42 different resistant patterns, grouped by antibiotic group, were found in dog and cat strains (Table 2). For both dogs and cats, the most prevalent was PEN alone (n = 11 and n = 9, respectively). The next most prevalent patterns in dogs were PEN-FOL (n = 9) and PEN-CEPHA (n = 7), followed by PEN-CEPHA-FOL (n = 5) and AMI-PEN-QUIN-CEPHA-CARB (n = 5). In contrast, for cats, the following most prevalent patterns were QUIN alone (n = 6), CEPHA alone (n = 5) and PEN-AMI-QUIN-CEPHA-FOL-CARB (n = 5). Although the results showed multiple AMR patterns, it is necessary to highlight that 73.8% (31/42) of them included resistance to the penicillins group, the most commonly used group of antibiotics in companion animals and also the most commonly administered in the studied population. 

## 3. Discussion

The emergence of AMR and MDR has become a global public health threat. In fact, they have been considered as the best illustration of the One Health approach [33], as it has been shown that animals can act as a reservoir of AMR genes and disseminate them in the environment [34]. This is of vital importance as humans nowadays maintain intimate contact with their companion animals, which poses a great danger to public health as pets could share their commensal microbiota with humans and thus the resistance genes that this microbiota possesses, leading to therapeutic failures in human and animal medicine [35]. In fact, the EMA considered that, although it is difficult to demonstrate the direction of AMR spread between animal and human bacteria, companion animals could be a reflection of AMR circulating in the household and therefore valuable information on AMR present in their owners [36]. However, few studies have addressed the prevalence of AMR and MDR in strains isolated from healthy companion animals in the EU, as most studies focus on strains isolated from diseased animals [37,38,39,40]. For this reason, it is necessary to assess the AMR and MDR present in companion animals (dogs and cats) commensal *E. coli*, in order to determine the impact that these resistances have on public health [41]. The present study demonstrates that 88.2% of the dogs studied and 72.6% of the cats studied in the Valencia Region were resistant to at least one of the 22 antibiotics of importance in public health, and 47.1% and 34.7% presented MDR, respectively. In addition, AMR was found against all antibiotics studied. 

A recent study conducted in healthy animals in Hangzhou (China) showed high percentages of MDR in *E. coli* strains isolated from dogs and cats, 41% and 30%, respectively [42]. However, studies addressing the situation of AMR and MDR in asymptomatic companion animals are scarce in the EU, as the main focus is usually on diseased animals. For example, in the first report published from the Chinese Companion Animal Antimicrobial Resistance Surveillance Network (CARPet), addressing the AMR of the most common bacteria isolated from infectious process of dogs and cats, showed that the total strains isolated of *E. coli* presented 51% of MDR [43]. Comparing these results with those of other studies from different geographical areas, such as some from the United States, showing high AMR (61.7%) and MDR (47.4%) in dogs [44], studies conducted in Chile where 96.9% of the dog strains studied showed AMR [45], or other studies carried out in Malaysia, where higher levels of MDR were found in dogs (72%) and cats (70%) [40], further highlights the global problem that public health worldwide is facing. Moreover, regardless of whether they were healthy or diseased animals, dogs generally showed higher percentages of MDR than cats. One hypothesis to explain these results could be due to the fact that dogs go to public spaces with their owners and share the environment with more people and animals [46], which may lead to the spread and acquisition of AMR genes, whereas cats have less interaction with the outside environment and only interact with their owners at home.

Regarding the AMR observed to each antibiotic, three antibiotics from the penicillins group were those with the highest AMR in the present study: AMP, TIC and AMC. The first was AMP, the antibiotic with the highest resistances in dogs, and the second in cats. These results agree with those reported in other studies carried out in Europe, with the results from Italy being the most similar to those of the present study [30]. Other authors have also reported the highest AMR to this antibiotic, ranging from 40 to almost 100% in different studies worldwide, regardless of whether they were studies carried out in asymptomatic [47,48,49,50] or symptomatic [43,44,51,52,53,54] dogs and cats. Moreover, these results have also been found in food-producing animals [17] and humans [51,55], which highlights the global impact of this issue. For TIC, the second most resistant antibiotic in our study in dogs and the first in cats, similar results have been found in other studies that showed around 50% of AMR [44,56,57]. Finally, regarding AMC, our high prevalence differs from that in other published studies, in which the AMR ranges from 5.4 to 45% [43,44,54,56,58]. On the contrary, AMR against AMC in diseased cats isolates were significantly lower than in dogs, according to other studies [43,54]. This could be explained because the 67.6% of the dogs vs. the 43.1% of the cats had been previously treated with antibiotics from the penicillins group in their lifetime, which may have favoured the occurrence of AMR in the study population. Moreover, it is important to highlight that penicillins, in particular AMP and AMC, are widely used and one of the first therapeutic options in urinary tract infections caused by *E. coli* [59]. However, the emergence of extended-spectrum β-lactamases (ESBL), strains of certain pathogens (especially *E. coli* and *Klebsiella pneumoniae*) that are resistant to β-lactam antibiotics (penicillins, cephalosporins and carbapenemases), are becoming more frequent [60]. For that reason, their detection is very important to guide appropriate treatment of infections and thus implement infection control measures and prevention of the spread of these resistant bacteria in healthcare facilities [61].

Following the AMR levels observed, TRS showed a higher resistance level in dogs than in cats, in line with previous studies [43,62,63]. These results should be widely monitored, as resistances to this antibiotic in AMR monitoring programmes in food-producing animals are among the highest in Europe [17], and the International Society for Companion Animal Infectious Diseases (ISCAID) recommends the use of TRS as first-line empirical treatments in urinary tract infections [58]. Regarding cephalosporins, similar results have been observed in dogs and cats, ranging from 16% to 50% among the different antibiotics evaluated in previous studies [64,65,66,67]. The phenotypic resistances observed to these β-lactams antibiotics (especially to 3rd and 4th gen. cephalosporins; CEP, CIX, CTA, and CTZ) also suggest that some of these strains could be ESBL-producing *E. coli* [66,67,68]. However, further molecular analyses are needed to detect the resistance genes of these strains not only phenotypically but also genotypically [69]. It is also important to highlight the resistance observed to 3rd and 4th gen. cephalosporins, similar to that observed for the quinolones group, as these CIAs should be the last ones used in veterinary when the prescription order is followed, as they belong to the last EMA category approved for use in veterinary medicine (Category B). Nevertheless, they are widely administrated according to different studies, especially for urinary infections in cats and dental infections in dogs [32,70,71].

In addition, four Category A antibiotics [19] were tested in this study, and a high percentage of AMR was found, considering that they are used as last resort antibiotics in humans. It is important to highlight the impact of these results due to the importance of these antibiotics in human medicine, as they are the last therapeutic option when all other antibiotic treatments have failed [72]. Regarding piperacillin/tazobactam (PIT), a high prevalence of AMR was found, mainly in dogs. Different results have been found in other studies in cats and dogs, 0% [57], or in humans, 7–12% [73,74]. PIT is a combination used to treat complicated infections in hospitals, including those caused by ESBL strains; therefore, the increasing AMR against this antibiotic could lead to an increase in hospital deaths among elderly, children or immunosuppressed patients [75]. 

Moreover, two of the Category A antibiotics studied were carbapenemases: ertapenem (ETP) and meropenem (MER). To the authors’ knowledge, this is the first study addressing the AMR observed against ETP in commensal *E. coli* in dogs and cats showing high resistance rates, although a case of ETP-resistant *Klebsiella pneumoniae* has been reported in dogs in Belgium [76]. Some other studies also address AMR to this antibiotic in *E. coli* isolated from calves [77], dairy cattle [78] and humans [79], which may be one of the sources of transmission of AMR against ETP to companion animals. Regarding MER, a low AMR range has been observed in different studies, between 3.5 and 6% [43,80,81]. But these results are more alarming, because in some studies MER resistances in human *E. coli* have been found to be 0% [73], posing a serious risk to humans in the transmission of AMR, leading to therapeutic failures in bacterial infections [82]. Finally, Tigecycline (TGC) is an antibiotic also used to treat complicated infections and should be reserved for infections caused by MDR bacteria when other treatment options are more toxic or less effective [83]. This may be the reason why it is the antibiotic with the least resistance observed in our study, in line with the results found in different studies in companion animals [74,84]. 

The present study revealed the importance of monitoring AMR and MDR trends in companion animals, as they could pose a direct threat to public health due to the spread of AMR genes. These results provide valuable information as a starting point and highlight the need for a One Health approach to implement new strategies in veterinary medicine for companion animals to control the alarming increase in AMR. However, more studies are needed to further study AMR in companion animals, not only phenotypically but also genotypically, as a more comprehensive analysis of this issue could help the scientific community better understand the dynamics of AMR genes between commensal and pathogenic bacteria from animals to humans and vice versa.

## 4. Materials and Methods

### 4.1. Experimental Design

The animal study was reviewed and approved by the Animal Ethics Committee of UCH-CEU University (research permit nº. CEEA 22/04). 

Veterinary Hospitals (VH) and Veterinary Clinics (VC) distributed throughout the Valencia Region were asked to voluntarily participate in this study. Of these, three VH and five VC were willing to cooperate. Thus, eight veterinary centres agreed to collaborate voluntarily. Three are characterised by being three large reference VH, where cases from the entire Valencia Region are referred, and five VC distributed throughout the region of Valencia also participated. 

### 4.2. Epidemiological Data Collection

To gather epidemiological data on the animals sampled, an epidemiological questionnaire was filled in for each animal. The questionnaire was divided into 3 parts. The first section referred to information on the source of the animals (origin details), and included the informed consent signed by the owners. The second included general information on the animal: sex, age and whether other animals cohabited at home. To categorise the age range into groups, dogs were classified as puppies and young dogs (≤2 years), mature dogs (3–7 years), and senior and geriatric dogs (≥8 years) [85]. In contrast, cats were classified as kittens and young cats (≤1 year), mature cats (2–8 years), and senior and geriatric cats (≥9 years) [86]. Finally, the third part included clinical data on the animal: whether the animal has any chronic disease and whether it takes any daily medication, and lastly, when the animal was last treated with antibiotics and which antibiotics it has taken throughout its life (questionnaire in Supplementary Materials; Part A). Data on dogs and cats were analysed separately.

### 4.3. Sample Collection

Between October 2022 and June 2023, samples from companion animals (dogs and cats) were taken in order to isolate commensal *E. coli*. To assess the antimicrobial profile, a rectal swab was taken from asymptomatic animals by introducing a swab into the rectum approximately 3 cm [87,88], using sterile cotton swabs (Cary-Blair sterile transport swabs, DELTALAB, Barcelona, Spain). Before taking the samples, the veterinarians examined the animals to make sure they had no disease symptoms. In addition, they took their vital signs to ensure that they were within normal ranges and were therefore considered as asymptomatic healthy animals. All samples were transported refrigerated at ≤4 °C to the microbiology laboratory at the Faculty of Veterinary Sciences of the University CEU Cardenal Herrera for microbial analyses within 24 h of collection.

### 4.4. E. coli Isolation

Rectal swabs were pre-enriched in buffered peptone water (BPW; Scharlau, Barcelona, Spain), in 1:10 vol/vol proportion, and incubated at 37 ± 1 °C for 24 ± 2 h. All the pre-enriched samples were inoculated onto selective culture agar for *E. coli* identification, Tryptone Bile X-glucuronide agar (TBX; Scharlau, Barcelona, Spain), and incubated at 37 ± 1 °C for 24 h. After incubation, colonies with compatible morphology with *E. coli* were selected and inoculated into a nutrient agar plate (Scharlau, Barcelona, Spain) and incubated at 37 ± 1 °C for 24 h. Finally, a biochemical test was performed to confirm *E. coli* (API-20E test, bioMerieux, Marcy l’Étoile, France). 

### 4.5. Antimicrobial Susceptibility Testing

To establish the epidemiological situation, an antimicrobial susceptibility test was carried out with antibiotics of importance in public health. AMR was evaluated using Minimum Inhibition Concentration (MIC) assay in EUGNF Gram Negative Sensititre Plate (Thermo Scientific™ Sensititre™ Plates, Madrid, Spain). Finally, Sensititre plate results were interpreted according to the European Committee on Antimicrobial Susceptibility Testing (EUCAST) established breakpoints 2022, available on the European Society of Clinical Microbiology and Infectious Diseases website (https://www.eucast.org/ast_of_bacteria/calibration_and_validation. Accessed on 12 September 2023). To detect AMR microorganisms, the phenotypic resistance should be studied in vitro. When the microorganism under study shows acquired resistance to at least one agent from three or more antimicrobial classes, it is defined as an MDR microorganism [11].

To this end, each bacterium was incubated in nutrient agar for 24 h at 37 ± 1 °C, after which colonies were transferred to 5 mL of sterile demineralised water (T3339; ThermoFisher Scientific, Madrid, Spain). The suspensions were mixed and adjusted, adding colonies until a 0.5 McFarland score was reached using a Nephelometer (Sensititre™ Nephelometer, ThermoFisher Scientific, Madrid, Spain). Then, 30 μL of this suspension were added to a vial containing 11 mL of Mueller–Hinton broth (T3462; ThermoFisher Scientific, Madrid, Spain) and mixed. From that suspension, 50 μL of the vial contents were transferred into each well of the Sensititre plate. After inoculation, the plates were sealed with plate film and incubated at 37 ± 1 °C for 24 h. The plates were read manually using a Sensititre Vizion (Thermo Scientific™ Sensititre™ Vizion™ Digital MIC Viewing System, ThermoFisher Scientific, Madrid, Spain).

To assess *E. coli* AMR profile, a commercial panel of 22 antibiotics with relevance in public health were used. The antibiotics are summarised in Table 3. 

Under the One Health concept, this commercial Sensititre plate was selected because 12 of these antibiotics belong to those that EARS-Vet wishes to monitor (AMC, AMP, PIT, GEN, CLE, CTA, CEP, TRS, CIP, TIG, ERT, and MER), and the other 10 belong to antibiotics used in human medicine (TIC, AMI, TOB, CXI, CUR, CIX, CTZ, LEV, NAL, and NIT).

### 4.6. Statistical Analysis

A Generalised Linear Model (GLM) using the probit link function, which assumed a binomial distribution for the influence of external factors in AMR and MDR patterns, was fitted to the data to determine whether there was an association with the categorical variables (animal origin, sex, whether or not the animal cohabits with other animals, whether or not the animal mixes with other animals outside, and clinical information related to whether or not the animal has any chronic disease or takes daily medication and when and with which antibiotics the animal has ever been treated). Also, for microbiological results, a GLM using the probit link function, which assumed a binomial distribution for AMR patterns in commensal bacteria of dogs, and a GLM using the probit link function, which assumed a binomial distribution for AMR patterns in commensal bacteria of cats, were performed. A *p*-value ≤ 0.05 was considered to indicate a statistically significant difference. Data are presented as least squares means ± standard error of the least squares means. Statistical analyses were carried out using the R software packages EMMs [89], car [90] and multicompView [91].

## 5. Conclusions

The results obtained in this study highlight the need to control the administration of antibiotics, not only in food-producing animals, but also in companion animals, which cohabit and interact with humans, domestic and wild animals and their environment, posing a risk in the dissemination of AMR and resistance genes. Furthermore, the similar AMR patterns observed between dogs and cats support the hypothesis of its transmission. However, no statistical differences were found between epidemiological groups, which may be more alarming, as AMR is widespread even if the animals have not been previously treated with antibiotics. These results are of special concern regarding CIAs, as high AMR levels are observed.

## Figures and Tables

**Table 1:** Comparison of antimicrobial resistance to commensal *Escherichia coli* per antibiotic group and per antibiotic tested between dogs and cats.

	Dog	Cat			Dog	Cat
Antibiotic Group	% AMR/Group	% AMR/Group	Antibiotic	EMA	% AMR/Antibiotic	% AMR/Antibiotic
**Aminoglycosides**	15.7 ^a^ ± 1.4	23.2 ^a^ ± 2.5	Amikacin	C	17.6 ^a,b,c^ ± 3.8	23.2 ^a,b,c,d^ ± 4.3
			Gentamicin	C	8.8 ^c,g^ ± 2.8	21.1 ^a,b,c^ ± 4.2
			Tobramycin	C	20.6 ^a,b,f^ ± 4	25.3 ^b,c,d^ ± 4.5
**Carbapenemases**	5.9 ^b^ ± 1.2	9.5 ^b,c^ ± 2.1	Ertapenem	A	5.9 ^g^ ± 2.3	12.6 ^a,f^ ± 3.4
			Meropenem	A	5.9 ^g^ ± 2.3	6.3 ^f,g^ ± 2.5
**Cephalosporins**	22.4 ^c^ ± 4.1	21.7 ^a^ ± 1.6	Cefepime	B	15.7 ^a,c^ ± 3.6	17.9 ^a,b^ ± 3.9
			Cefixime	B	23.5 ^a,b,f,h^ ± 4.2	30.5 ^c,d,e^ ± 4.7
			Cefotaxime	B	28.4 ^b,f,h^ ± 4.5	16.8 ^a,b^ ± 3.8
			Cefoxitin	C	23.5 ^a,b,f,h^ ± 4.2	21.1 ^a,b,c^ ± 4.2
			Cefuroxime	C	21.6 ^a,b,f^ ± 4.1	18.9 ^a,b,c^ ± 4
			Cefalexin	C	23.5 ^a,b,f,h^ ± 4.2	24.2 ^b,c,d^ ± 4.4
			Ceftazidime	B	20.6 ^a,b,f^ ± 4	21.1 ^a,b,c^ ± 4.2
**Nitrofurans**	14.7 ^a^ ± 3.5	4.2 ^c,d^ ± 2.1	Nitrofurantoin	D	14.7 ^a^ ± 3.5	4.2 ^c,d^ ± 2.1
**Penicillins**	45.8 ^d^ ± 1.9	25.5 ^a^ ± 2.2	Ampicillin	D	62.7 ^d^ ± 4.8	38.9 ^e^ ± 5
			Amoxicillin/ Clavulanic acid	C	43.1 ^e^ ± 4.9	18.9 ^a,b,c^ ± 4
			Piperacillin/ Tazobactam	A	16.7 ^a,c^ ± 3.7	4.2 ^g^ ± 2.1
			Ticarcillin	D	60.8 ^d^ ± 4.8	40 ^e^ ± 5
**Quinolones**	23.9 ^c^ ± 2.1	25.6 ^a^ ± 3.8	Ciprofloxacin (FQ)	B	22.5 ^a,b,f^ ± 4.1	21.1 ^a,b,c^ ± 4.2
			Levofloxacin (FQ)	B	19.6 ^a,b,f^ ± 3.9	21.1 ^a,b,c^ ± 4.2
			Nalidixic acid (Q)	B	29.4 ^f,h^ ± 4.5	34.7 ^d,e^ ± 4.9
**Folate inhibitor pathway**	35.3 ^e^ ± 4.7	16.8 ^a,b^ ± 3.8	Sulfamethoxazole/Trimethoprim	D	35.3 ^e,h^ ± 4.7	16.8 ^a,b^ ± 3.8
**Glycylcycline**	2.9 ^b^ ± 1	1.1 ^d^ ± 1	Tigecycline	A	2.9 ^b^ ± 1	1.1 ^d^ ± 1

% AMR: percentage of antimicrobial resistance (per group and per antibiotic). FQ: fluoroquinolone. Q: quinolone. ^a–h^: different superscripts in each column indicate statistically significant differences (*p*-value ≤ 0.05) for the resistances obtained against the different antibiotics studied. ±: standard error EMA: European Medicines Agency. This column indicates the EMA categorisation of antibiotics used in animals to promote their responsible use in order to protect animal and public health.

**Table 2:** Number of dog and cat commensal *Escherichia coli* strains isolated resistant to the different number of antimicrobials tested and their antimicrobial resistance patterns by antibiotic groups.

N of AB Groups	n of Dog Isolates (%)	n of Cat Isolates (%)	N of Isolates (%)	AMR Patterns
**0**	-	-	12 (6.1%)	-
**1**	11 (10.8%)	9 (9.5%)	20 (10.2%)	PEN
	4 (3.9%)	5 (5.3%)	9 (4.6%)	CEPHA
	2 (2.1%)	6 (6.3%)	8 (4.1%)	QUIN
	-	3 (3.2%)	3 (1.5%)	AMINO
**2**	2 (2.1%)	-	2 (1.1%)	PEN-AMINO
	7 (6.9%)	3 (3.2%)	10 (5.1%)	PEN-CEPHA
	3 (2.9%)	3 (3.2%)	6 (3.0%)	PEN-QUIN
	9 (8.8%)	4 (4.2%)	13 (6.6%)	PEN-FOL
	1 (1.1%)	-	1 (0.5%)	CEPHA-NITRO
	1 (1.1%)	-	1 (0.5%)	CEPHA-QUIN
	2 (2.1%)	-	2 (1.1%)	QUIN-FOL
	-	1 (1.1%)	1 (0.5%)	ANIMO-FOL
	-	2 (2.1%)	2 (1.1%)	AMINO-QUIN
**3**	3 (2.9%)	2 (2.1%)	5 (2.5%)	PEN-AMINO-CEPHA
	1 (1.1%)	-	1 (0.5%)	PEN-AMINO-NITRO
	2 (2.1%)	2 (2.1%)	4 (2.0%)	PEN-AMINO-QUIN
	4 (3.9%)	4 (4.2%)	8 (4.1%)	PEN-CEPHA-QUIN
	5 (5.0%)	1 (1.1%)	6 (3.0%)	PEN-CEPHA-FOL
	2 (2.1%)	-	2 (1.1%)	PEN-NITRO-FOL
	4 (3.9%)	1 (1.1%)	5 (2.5%)	PEN-QUIN-FOL
	1 (1.1%)	1 (1.1%)	2 (1.1%)	PEN-QUIN-NITRO
	1 (1.1%)	-	1 (0.5%)	PEN-CARB-GLYC
	1 (1.1%)	1 (1.1%)	2 (1.1%)	AMINO-CEPHA-QUIN
**4**	3 (2.9%)	3 (3.2%)	6 (3.0%)	PEN-AMINO-CEPHA-QUIN
	2 (2.1%)	-	2 (1.1%)	PEN-AMINO-CEPHA-FOL
	1 (1.1%)	1 (1.1%)	2 (1.1%)	PEN-AMINO-QUIN-FOL
	2 (2.1%)	-	2 (1.1%)	PEN-AMINO-NITRO-FOL
	2 (2.1%)	-	2 (1.1%)	PEN-CEPHA-QUIN-FOL
	-	1 (1.1%)	1 (0.5%)	AMINO-CEPHA-QUIN-FOL
	-	1 (1.1%)	1 (0.5%)	AMINO-CEPHA-QUIN-CARB
**5**	5 (5.0%)	3 (3.2%)	8 (4.1%)	PEN-AMINO-CEPHA-QUIN-CARB
	1 (1.1%)	2 (2.1%)	3 (1.5%)	PEN-AMINO-CEPHA-QUIN-FOL
	-	1 (1.1%)	1 (0.5%)	PEN-AMINO-QUIN-FOL-CARB
	-	1 (1.1%)	1 (0.5%)	PEN-AMINO-CEPHA-NITRO-FOL
	1 (1.1%)	-	1 (0.5%)	PEN-CEPHA-QUIN-NITRO-CARB
	1 (1.1%)	-	1 (0.5%)	PEN-CEPHA-QUIN-NITRO-FOL
**6**	1 (1.1%)	1 (1.1%)	1 (0.5%)	PEN-AMINO-CEPHA-QUIN-NITRO-CARB
	2 (2.1%)	-	2 (1.1%)	PEN-AMINO-CEPHA-QUIN-NITRO-FOL
	-	5 (5.3%)	5 (2.5%)	PEN-AMINO-CEPHA-QUIN-FOL-CARB
	-	1 (1.1%)	1 (0.5%)	PEN-AMINO-CEPHA-QUIN-FOL-GLYC
**7**	1 (1.1%)	1 (1.1%)	2 (1.1%)	PEN-AMINO-CEPHA-QUIN-NITRO-FOL-CARB
**8**	2 (2.1%)	-	2 (1.1%)	PEN-AMINO-CEPHA-QUIN-NITRO-FOL-CARB-GLYC

N: total number. n: number. AB: antibiotics. AMR: antimicrobial resistance. PEN: penicillins. AMINO: aminoglycosides. CEPHA: cephalosporins. QUIN: quinolones. NITRO: nitrofurans. FOL: folate inhibitors pathways. CARB: carbapenemases. GLYC: glycylcyclines.

**Table 3:** Antibiotics and their concentrations of EUGNF Gram Negative Sensititre Plate (Thermo Scientific™ Sensititre™, Madrid, Spain).

Antibiotic Group	Antibiotic	Abbreviation	Concentration	EUCAST Breakpoints
**Aminoglycosides**	Amikacin	AMI	2–32 μg/mL	>8 μg/mL
	Gentamicin	GEN	0.5–8 μg/mL	>2 μg/mL
	Tobramycin	TOB	0.5–8 μg/mL	>2 μg/mL
**Carbapenemases**	Ertapenem	ERT	0.12–2 μg/mL	>0.5 μg/mL
	Meropenem	MER	0.12–16 μg/mL	>8 μg/mL
**Cephalosporins**	Cefepime	CEP	0.5–8 μg/mL	>4 μg/mL
	Cefixime	CIX	0.5–2 μg/mL	>1 μg/mL
	Cefotaxime	CTA	0.5–4 μg/mL	>2 μg/mL
	Cefoxitin	CXI	2–16 μg/mL	>8 μg/mL
	Cefuroxime	CUR	2–16 μg/mL	>8 μg/mL
	Cefalexin	CLE	8–32 μg/mL	>16 μg/mL
	Ceftazidime	CTZ	0.5–8 μg/mL	>4 μg/mL
**Nitrofurans**	Nitrofurantoin	NIT	32–64 μg/mL	>64 μg/mL
**Penicillins**	Ampicillin	AMP	2–16 μg/mL	>8 μg/mL
	Amoxicillin/ Clavulanic acid	AMC	2/2–32/2 μg/mL	>8 μg/mL
	Piperacillin/ Tazobactam	PIT	2/4–32/4 μg/mL	>8 μg/mL
	Ticarcillin	TIC	4–32 μg/mL	>16 μg/mL
**Quinolones**	Ciprofloxacin (FQ)	CIP	0.12–1 μg/mL	>0.5 μg/mL
	Levofloxacin (FQ)	LEV	0.25–2 μg/mL	>1 μg/mL
	Nalidixic acid (Q)	NAL	16 μg/mL	>8 μg/mL
**Folate inhibitor pathway**	Sulfamethoxazole/Trimethoprim	TRS	1/19–8/152 μg/mL	>4 μg/mL
**Glycylcycline**	Tigecycline	TIG	0.5–4 μg/mL	>0.5 μg/mL

EUCAST: European Committee on Antimicrobial Susceptibility Testing. FQ: Fluoroquinolone. Q: Quinolone.

## Data Availability

Data is contained within the article and Supplementary Material.

## Supplementary Materials

The following supporting information can be downloaded at https://www.mdpi.com/article/10.3390/antibiotics12111638/s1, Part A: Questionnaire. Antimicrobial resistance study in companion animals.
