# Supplementary material for: Multidrug-Resistant Escherichia coli Strains to Last Resort Human Antibiotics Isolated from Healthy Companion Animals in Valencia Region
Source: Antibiotics (Basel). 2023 Nov 19;12(11):1638. doi: 10.3390/antibiotics12111638 (PMC10669260; doi:10.3390/antibiotics12111638)
Supplement: Supplementary file 1 [file antibiotics-12-01638-s001.zip › antibiotics-2683556-supplementary.pdf]

ANTIMICROBIAL RESISTENCE STUDY IN COMPANION ANIMALS

STUDY PRESENTATION

Antimicrobial resistance (AMR) is defined as the ability developed by microorganisms to tolerate antibiotic treatments. As a result, in both animal and human health, we are running out of effective tools to combat diseases and infections, posing a highly relevant issue today with direct impact on public health.

In recent years, due to the traditional, widespread, and inappropriate use of antibiotics in all areas of healthcare, the emergence of resistance has significantly increased. Furthermore, the transmission of these resistances among animals, humans, and the environment has been demonstrated. Therefore, it is crucial to always use them under the supervision of a healthcare professional, respecting the prescribed dosage and treatment duration.

With the aim of assessing the current situation of antibiotic resistance in companion animals, specifically in dogs and cats in the province of Valencia, we are conducting a study in which we will analyze various samples, both from healthy animals and from animals with an ongoing bacterial infectious process. To do this, we will need to collect a sample using a rectal swab. Additionally, a short questionnaire will be conducted to gather relevant data about the pets, which may influence the occurrence and transmission of AMR.

Thanking you in advance for your cooperation, best regards.

ANTIMICROBIAL RESISTENCE STUDY IN COMPANION ANIMALS

Animal ID (Dog/Cat and Name/Case number): ____

Name of the Veterinary Center: ____

Sample collection and questionnaire execution date (yyyy/MM/dd):___________________________________

INFORMED CONSENT

When filling out the following survey, I declare:

a) That I have been sufficiently informed about:

- i. The objectives of the research project, as well as the methodology to be used in it.
- ii. The role my pet plays in the research project.
- iii. The use that will be made of the information obtained through my pet's collaboration.

b) That my participation is entirely voluntary and free, and that I can withdraw from the study at any time.

c) That I have had the opportunity to ask questions related to the study, and I have received and understood the relevant explanations.

d) That I have full legal representation or legitimate rights to represent my pet.

e) That I have read and understand the contents of this document, comprehend the commitments I am making, and expressly accept them

Full name of the owner and DNI (Spanish identification number):

____

Animal name and microchip number:____

Address and postal code:____

Owner’s signature:

ANTIMICROBIAL RESISTENCE STUDY IN COMPANION ANIMALS

GENERAL PET INFORMATION

Age of the pet:_____________________

Sex:
- ☐ Female
- ☐ Male

¿ Does the pet live with other animals?:
- ☐ Yes* - If affirmative, which other animals?: ____
- ☐ No

Does the pet have relationship with other animals outside the home?:
- ☐ Yes
- ☐ No

PET CLINICAL DATA

Does the pet have any chronic disease?:
- ☐ Yes - If affirmative, which disease?: ____
- ☐ No

Does the pet take any daily medication?:
- ☐ Yes - If affirmative, which medication?:____
- ☐ No

When was the last time the pet was treated with an antibiotic?:
- ☐ Currently
- ☐ More than a month ago, but less than six months
- ☐ More than six months ago
- ☐ Never

Indicates with which antibiotic/antibiotics the animal has been treated throughout its life:

____

____

____
